# Supplementary material for: Asymmetric localization of DLC1 defines avian trunk neural crest polarity for directional delamination and migration
Source: Nat Commun. 2017 Oct 30;8:1185. doi: 10.1038/s41467-017-01107-0 (PMC5662599; doi:10.1038/s41467-017-01107-0)
Supplement: Supplementary file 1 — Supplementary Information [file 41467_2017_1107_MOESM1_ESM.pdf]

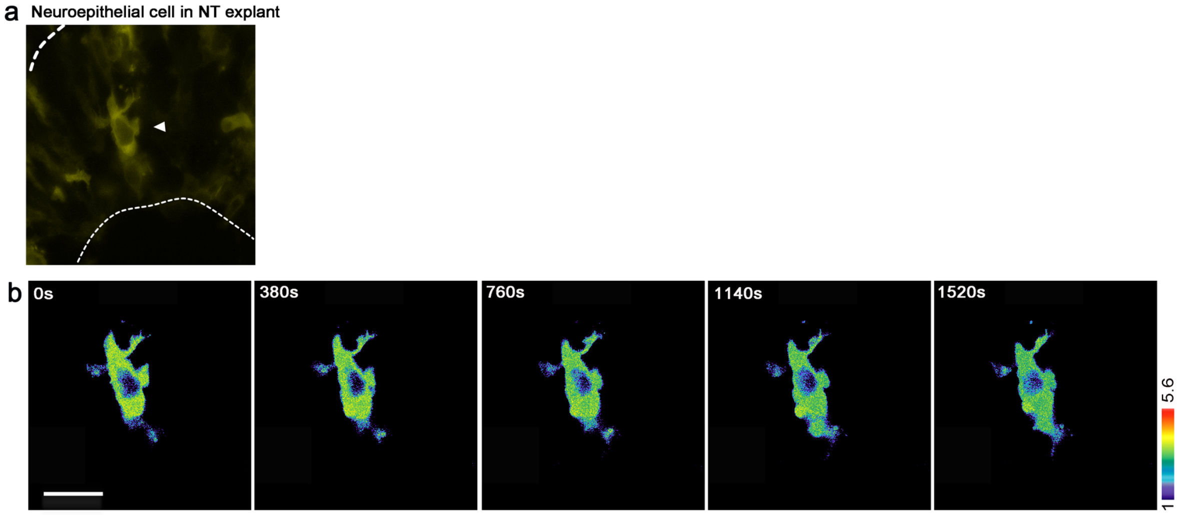

Supplementary Figure 1. Moderate level of RHOA activity is distributed throughout the cytoplasm of neuroepithelial cell. (a) White dotted lines outline the border of the neural tube (NT) explant. White arrowhead indicates the representative neuroepithelial cell shown at high magnification in (b). (b) Processed FRET signal images of time lapse-series of a neuroepithelial cell electroporated with the FRET probe for RHOA. n=70/15 explants. Scale bar, 20μm.

a

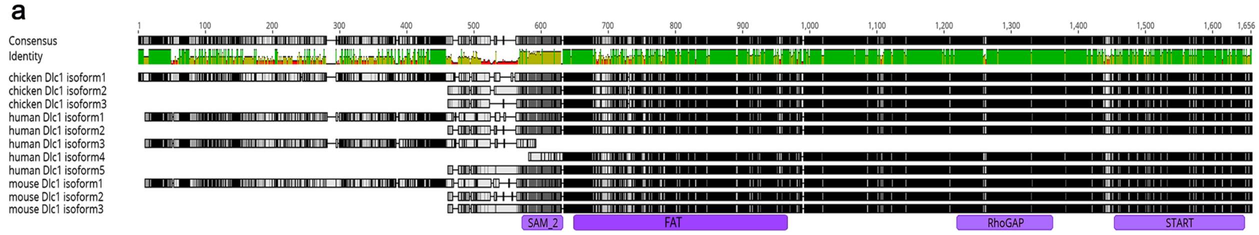

b

	mouse	human	chicken
mouse Dlc1 isoform1		83.581%	72.133%
human Dlc1 isoform1	83.581%		74.288%
chicken Dlc1 isoform1	72.133%	74.288%	

	mouse	human	chicken
mouse Dlc1 isoform3		92.635%	84.431%
human Dlc1 isoform5	92.635%		84.417%
chicken Dlc1 isoform2	84.431%	84.417%	

	mouse	human	chicken
mouse Dlc1 isoform2		92.681%	84.495%
human Dlc1 isoform2	92.681%		84.206%
chicken Dlc1 isoform3	84.495%	84.206%	

Supplementary Figure 2. Amino acid sequence alignment of DLC1 isoforms between chick, mouse and human. (a) Conserved sequences are highlighted in green and the SAM, FAT, RhoGAP and START domains are boxed in purple. The proteins compared are: Chick DLC1 isoform 1 (XP_420693), Chick DLC1 isoform 2 (XP_004936066), Chick DLC1 isoform 3 (XP_004936068), mouse DLC1 isoform 1 (NP_001181869), mouse DLC1 isoform 2 (NP_056617), mouse DLC1 isoform 3 (NP_001181870), human DLC1 isoform 1 (NP_872584), human DLC1 isoform 2 (NP_006085), human DLC1 isoform 3 (NP_079043), human DLC1 isoform 4 (NP_001157743) and human DLC1 isoform 5 (NP_001303597). (b) Tables indicate the percentage of amino acid sequences identity of DLC1 isoforms between mouse, human and chicken.

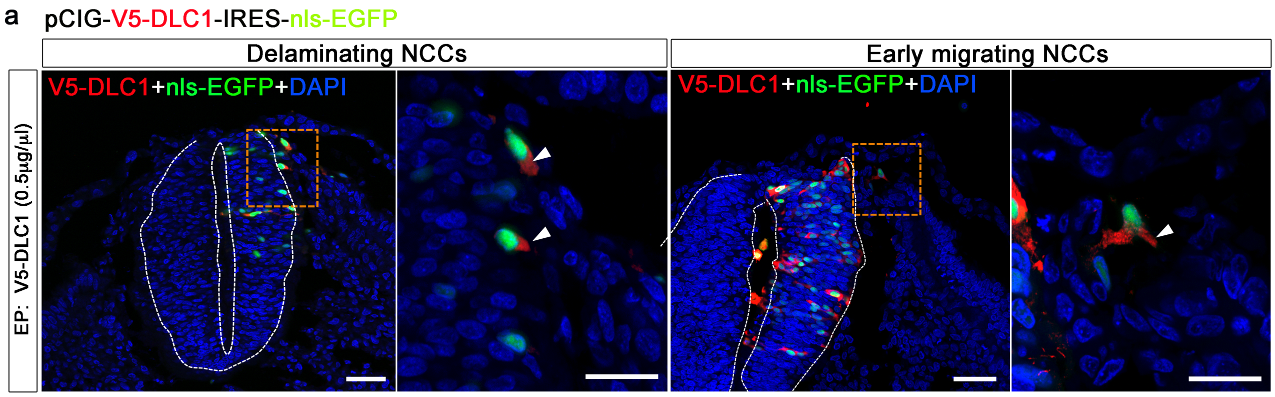

Supplementary Figure 3. Asymmetric localization of ectopic DLC1 expression in delaminating and early migrating NCCs. (a) Electroporation (EP) of V5-tagged DLC1 in pCIG-IRES-nls-EGFP vector at low concentration (0.5µg/µl) and analyze 24 hpt for immunofluorescence with GFP and V5 antibodies on transverse sections of electroporated embryos (n=10). Scale bars, 20µm. The dashed boxes indicate the regions shown at higher magnification. Scale bars, 50µm. White arrowheads indicate transfected NCCs with nuclear GFP expression (green) and asymmetric localization of ectopic DLC1 protein in the cytoplasm (red). White dotted lines outline the border of the neural tube and its lumen.

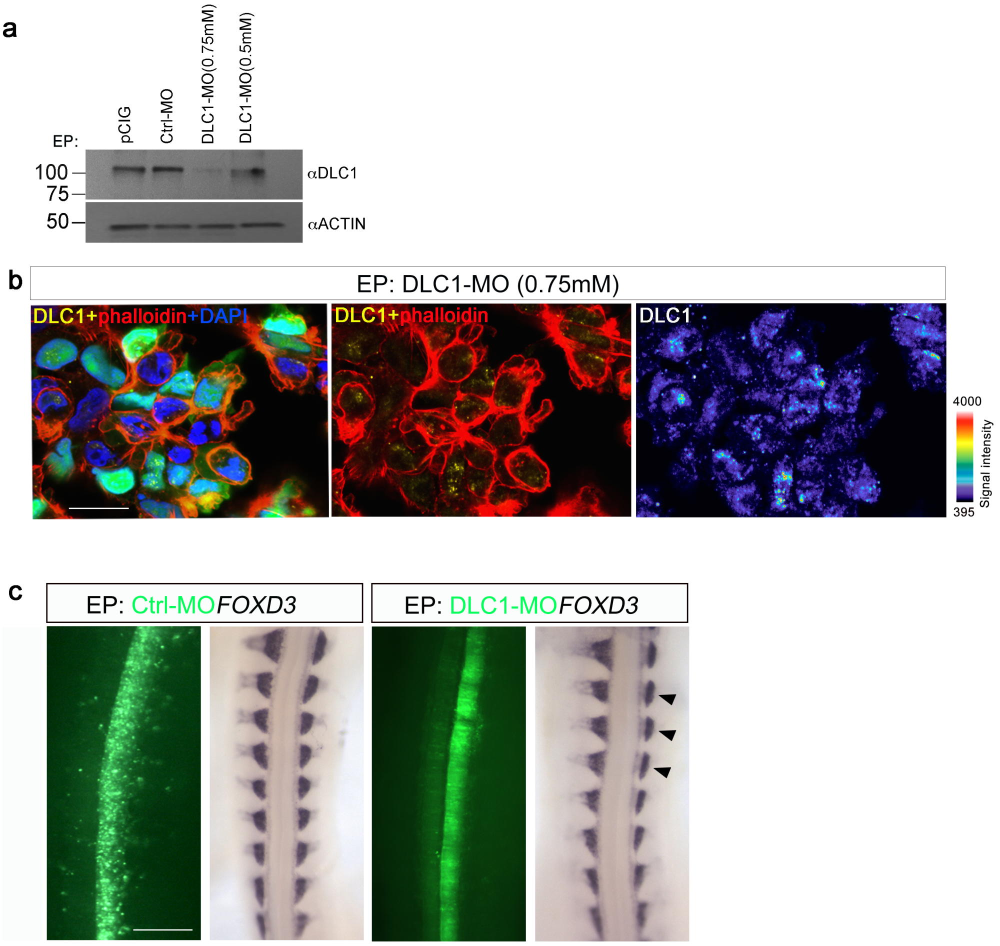

Supplementary Figure 4. DLC1 is required for neural crest delamination. (a) Western blot analysis of lysate from chick neural tubes electroporated with the indicated MOs and actin as loading control. (b) Immunofluorescence for DLC1 and phalloidin to label actin in cultured NCCs expressing DLC1-MO (0.75mM) and nuclei are stained with DAPI. Scale bar: 50µm. (c) In situ hybridization of *FOXD3* in embryos electroporated with Ctrl-MO (n=6) or DLC1-MO (n=7). Black arrowheads indicate reduction in the amount *FOXD3*$^{+}$ migratory NCCs in the transfected side of DLC1-MO-treated embryo. Scale bar, 10µm.

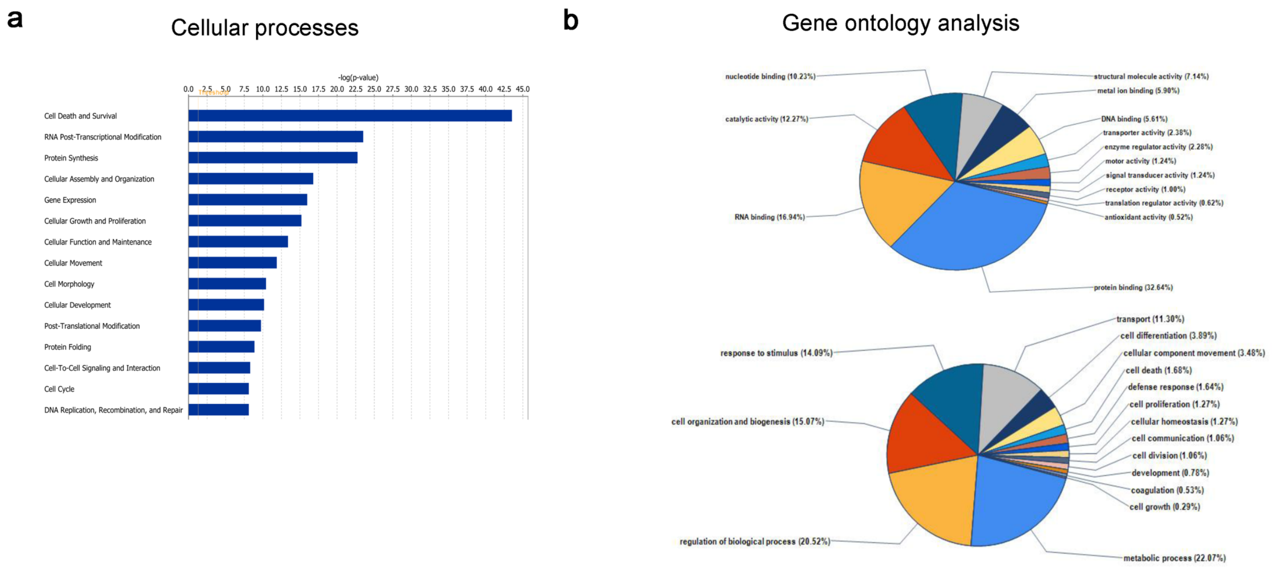

Supplementary Figure 5. Functional classification of proteins enriched by DLC1. (a) Cellular processes association of altered proteins. (b) Gene ontology analysis of proteins identified from DLC1-pull down fraction based on annotation with reference to human unipot protein database.

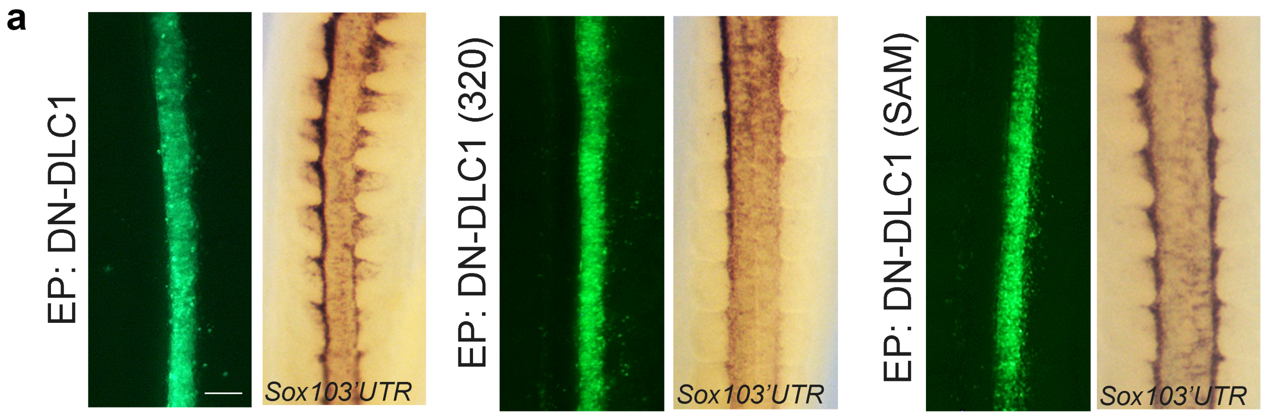

Supplementary Figure 6. Overexpression of DN-Dlc1 or DN-Dlc1 (320) but not DN-Dlc1 (SAM) reduced NC delamination. **(a) In situ hybridization of *Sox103'UTR* in embryos electroporated with DN-Dlc1 (n=8), DN-Dlc1 (320) (n=7), DN-Dlc1 (SAM) (n=9) at 24 hpt. Scale bar, 10μm.**

a

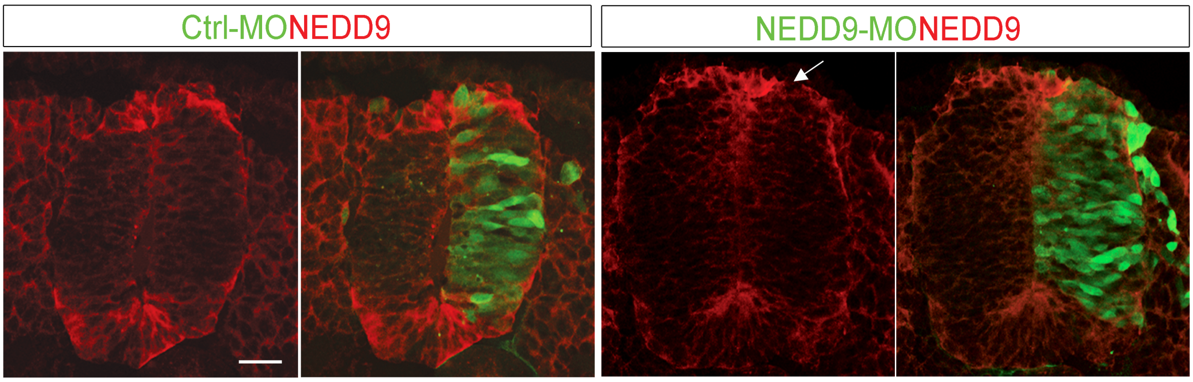

Supplementary Figure 7. Downregulation of NEDD9 protein expression in cells expressing NEDD9-MO. (a) Immunofluorescence for NEDD9 on transverse sections of embryos electroporated with Ctrl-MO (n=5) and NEDD9-MO (n=5). White arrow indicates reduced NEDD9 protein expression in delaminating NCCs expressing NEDD9-MO. Scale bar, 50μm.

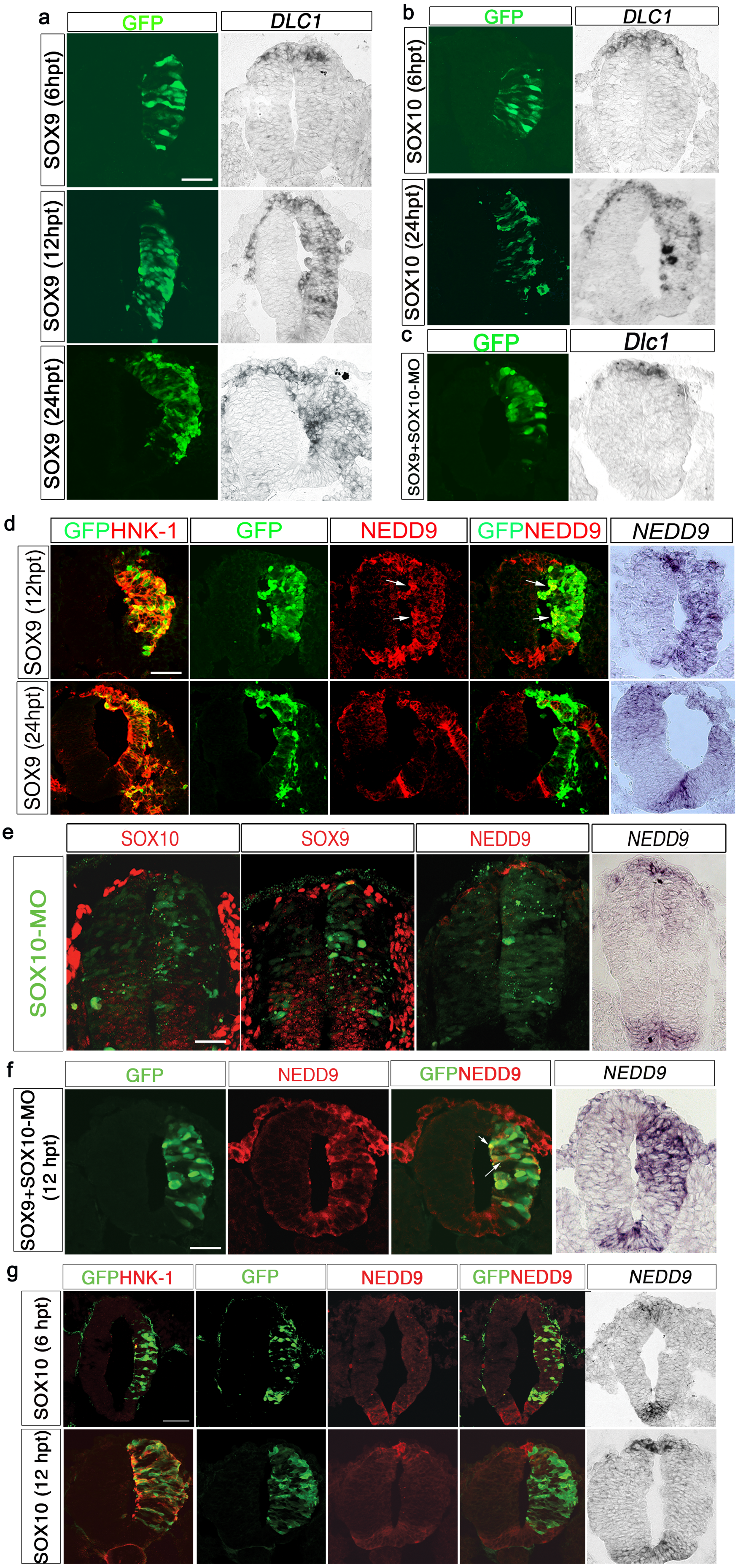

a
GFP
DLC1
SOX9 (6hpt)
SOX9 (12hpt)
SOX9 (24hpt)
b
GFP
DLC1
SOX10 (6hpt)
SOX10 (24hpt)
c
GFP
Dlc1
SOX9+SOX10-MO
d
GFPHNK-1
GFP
NEDD9
GFPNEDD9
NEDD9
SOX9 (12hpt)
SOX9 (24hpt)
e
SOX10
SOX9
NEDD9
NEDD9
SOX10-MO
f
GFP
NEDD9
GFPNEDD9
NEDD9
SOX9+SOX10-MO (12 hpt)
g
GFPHNK-1
GFP
NEDD9
GFPNEDD9
NEDD9
SOX10 (6 hpt)
SOX10 (12 hpt)

Supplementary Figure 8. SOX10 and Sox9 regulate *DLC1* and *NEDD9* expression respectively. In situ hybridization for *DLC1* on transverse sections of embryos electroporated with SOX9 at 6 hpt (n=6), 12 hpt (n=7) and 24 hpt (n=7) (a) or SOX10 (b) at 6 hpt (n=5) and 24 hpt (n=6). (c) In situ hybridization for *DLC1* on transverse sections of embryo electroporated with SOX9+SOX10-MO at 24 hpt (n=7). Scale bar, 50μm. (d) Immunofluorescence for HNK-1, NEDD9 and in situ hybridization for *NEDD9* on transverse sections of embryos electroporated with SOX9 at 12 (n=5) and 24 hpt (n=5). White arrows indicate cell-autonomous induction of NEDD9 in SOX9 expressing cells. (e) Immunofluorescence for SOX10, SOX9, NEDD9 and in situ hybridization for *NEDD9* on transverse sections of embryos electroporated with SOX10-MO at 24 hpt (n=5). (f) Immunofluorescence for NEDD9 and in situ hybridization for *NEDD9* on transverse sections of embryos electroporated with SOX9+SOX10-MO at 12 hpt (n=5). (g) Immunofluorescence for HNK1, NEDD9 and in situ hybridization for *NEDD9* on transverse sections of embryos electroporated with SOX10 at 6 (n=5) and 12 hpt (n=5). Scale bars, 50μm.

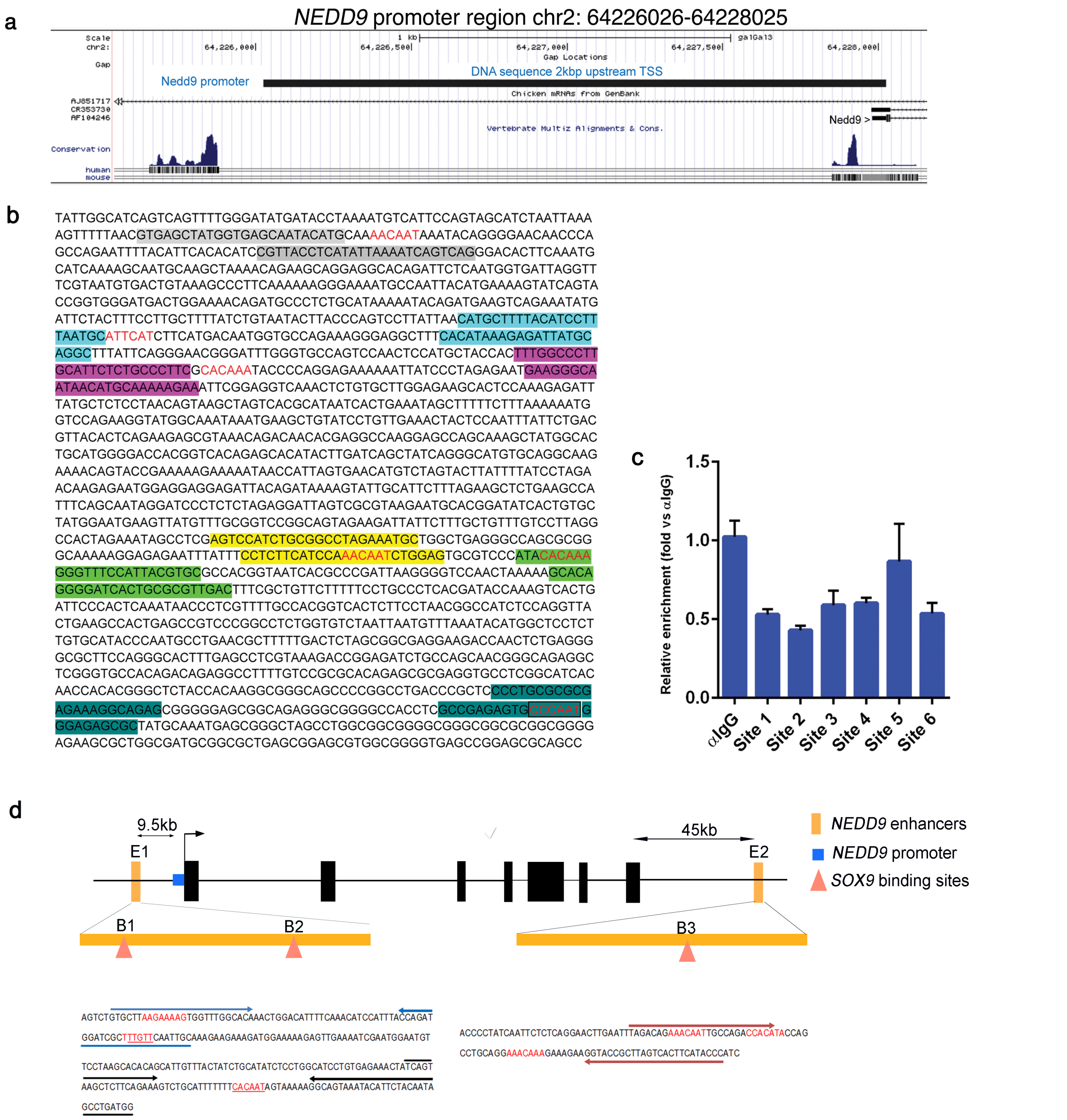

Supplementary Figure 9. SOX9 does not bind to the *NEDD9* promoter region. (a) Schematic representation showing the genomic position of a 2 kb chick *NEDD9* promoter region upstream from its transcription start site (TSS). Peaks indicate degree of sequence conservation of chick *NEDD9* promoter with human and mouse counterparts. (b) Nucleotide sequence of a 2 kb chick *NEDD9* promoter contains 6 putative *SOX9* binding motifs (red) flanked by two complementary primer binding sites for ChIP-qPCR (site1: grey; site 2: blue; site 3, pink; site 4, yellow; site 5, green; site 6, dark green). (c) Fold enrichments of two independent ChIP-PCR assays for SOX9 and IgG (negative control) on 6 putative *SOX9* binding motifs within 2kb *NEDD9* promoter region. Data represented as fold enrichments for putative *SOX9* binding motifs relative to the control. (d) Nucleotide sequence of chick *NEDD9* enhancers E1 and E2. Colored arrows indicate primer direction for the detection of *SOX9* binding motifs (red) by ChIP-qPCR (B1: blue arrows; B2: black arrows; B3; red arrows).

Fig. 3b

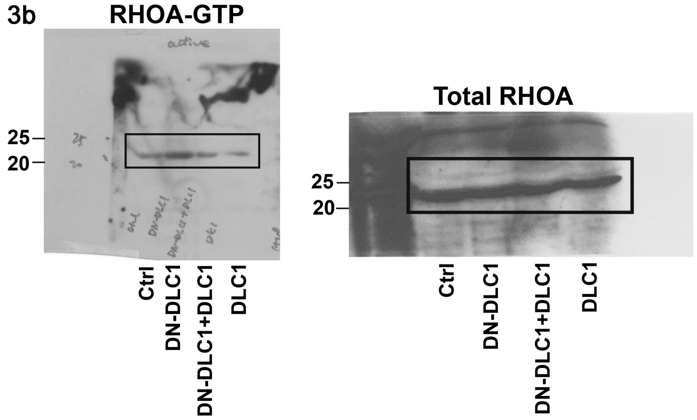

Fig. 5d

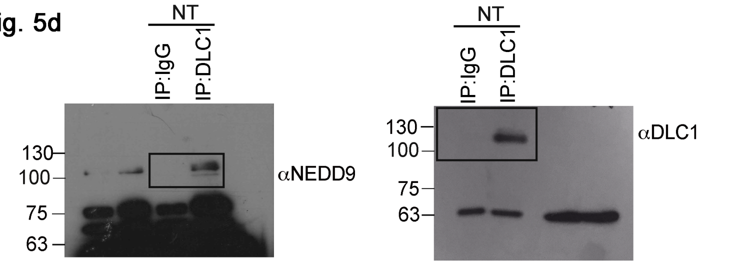

Fig. 5e

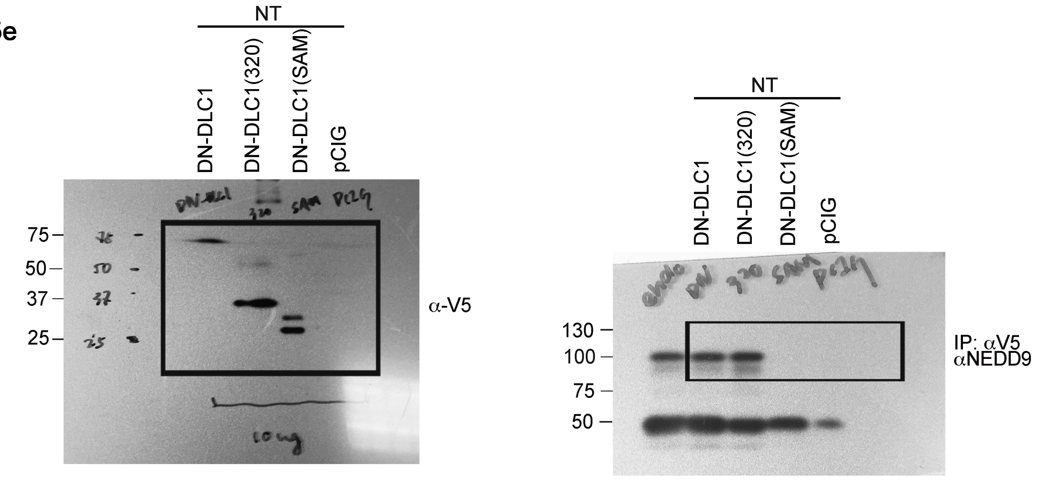

Supplementary Fig. 4a

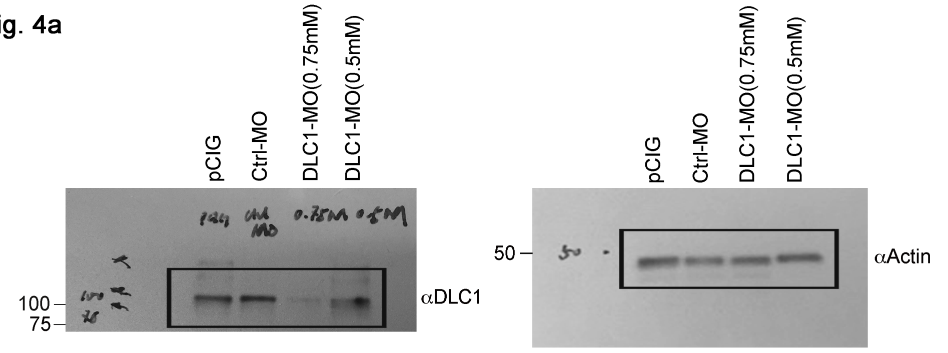

Supplementary Figure 10. Uncropped Western Blot images. The corresponding main and supplementary figures are shown. Black boxes highlight the cropped segment presented in main and supplementary figures.
