# Supplementary material for: Asymmetric localization of DLC1 defines avian trunk neural crest polarity for directional delamination and migration
Source: Nat Commun. 2017 Oct 30;8:1185. doi: 10.1038/s41467-017-01107-0 (PMC5662599; doi:10.1038/s41467-017-01107-0)
Supplement: Supplementary file 3 — Description of Additional Supplementary Files [file 41467_2017_1107_MOESM3_ESM.docx]

**Description of Additional Supplementary Files**

File Name: Supplementary Data 1

Description: Dlc1 interacting proteins are classified based on their functions in different biological processes

File Name: Supplementary Data 2

Description: Dlc1 interacting proteins are associated with different signaling pathways as assessed by Ingenuity Pathway Analysis.

File Name: Supplementary Movie 1

Description: Polarized RhoA activity in a neural crest cell undergoing directional migration. These movie files contains 1862 second (31 min) imaging sequences with 38 seconds intervals between frames, corresponding to the images shown in figure 1e. White arrowheads indicate dynamic RhoA activity in membrane protrusion of the leading edge.

File Name: Supplementary Movie 2

Description: Re-orientation of asymmetry RhoA signal in a neural crest cell undergoing a change in migratory direction in response to SDF-1. This movie file contains 3990 second (66.5 min) imaging sequences with 38 seconds intervals between frames, corresponding to the images shown in figure 1i.

File Name: Supplementary Movie 3

Description: Dynamics of RhoA signal in a neural crest cell undergoing front to back switch in response to SDF-1. This movie file contains 3724 second (62 min) imaging sequences with 38 seconds intervals between frames, corresponding to the images shown in figure 1l.

File Name: Supplementary Movie 4

Description: Protrusion dynamics and migrator behavior of delaminating neural crest cell expressing GFP vector control and Lifeact-mCherry. This movie file contains 40 min imaging sequences with 1 min intervals between frames, corresponding to the images shown in figure 4a.

File Name: Supplementary Movie 5

Description: Protrusion dynamics and migratory behavior of delaminating neural crest cell overexpressing Dlc1 and Lifeact-mCherry. These movie files contain 40 min imaging sequences with 1 min intervals between frames, corresponding to the images shown in figure 4a.

File Name: Supplementary Movie 6

Description: Protrusion dynamics and migratory behavior of delaminating neural crest cell overexpressing DN-Dlc1 and Lifeact-mCherry. These movie files contain 40 min imaging sequences with 1 min intervals between frames, corresponding to the images shown in figure 4a.

File Name: Supplementary Movie 7

Description: Polarized RhoA activity in a neural crest cell expressing vector control and undergoing directional migration toward SDF-1. These movie files contain 1862 second (~31mins) imaging sequences with 38 seconds intervals between frames, corresponding to the images shown in figure 4h.

File Name: Supplementary Movie 8

Description: Low and lack of polarized RhoA activity in a neural crest cell overexpressing Dlc1 and undergoing defective movement even in the presence of SDF-1. These movie files contain 1862 second (~31mins) imaging sequences with 38 seconds intervals between frames, corresponding to the images shown in figure 4h.

File Name: Supplementary Movie 9

Description: High and lack of polarized RhoA activity in a neural crest cell overexpressing DN-Dlc1 and undergoing defective migration even in the presence of SDF-1. These movie files contain 1862 second (~31mins) imaging sequences with 38 seconds intervals between frames, corresponding to the images shown in figure 4h.

File Name: Supplementary Movie 10

Description: Aberrant distribution of RhoA activity around the nucleus of a neural crest cell overexpressing Nedd9-MO without discernible back-front polarity axis. These movie files contain 1862 second (31mins) imaging sequences with 38 seconds intervals between frames, corresponding to the images shown in figure 6c.

File Name: Supplementary Movie 11

Description: Restoration of polarized RhoA activity and directional migration in a neural crest cell overexpressing DN-Dlc1+Nedd9. These movie files contain 1862 second (31mins) imaging sequences with 38 seconds intervals between frames, corresponding to the images shown in figure 6c.

File Name: Supplementary Movie 12

Description: Polarized RhoA activity in a neural crest cell overexpressing Nedd9. These movie files contain 1444 second (24mins) imaging sequences with 38 seconds intervals between frames, corresponding to the images shown in figure 6c.
